# Patellar mobility and lower limb kinematics during functional activities in individuals with and without patellar tendinopathy

**DOI:** 10.1016/j.knee.2021.04.002

**Published:** 2021-05-03

**Authors:** Rondy Michael Lazaro, Richard B. Souza, Anthony C. Luke

**Affiliations:** a Department of Physical Medicine and Rehabilitation, University of Rochester, 601 Elmwood Ave., Box 664, Rochester, NY 14642, USA; b Department of Physical Therapy and Rehabilitation Science, University of California, San Francisco, 185 Berry Street, Suite 350, San Francisco, CA 94107, USA; c Department of Orthopaedic Surgery, University of California, San Francisco, Orthopaedic Institute, 1500 Owens St., San Francisco, CA 94158, USA

**Keywords:** Arthrometer, Kinematics, Hypermobility, Motion analysis, Patellar tendon

## Abstract

**Study design:**

Case-control.

**Objective:**

To examine whether patients with patellar tendinopathy (PT) display greater patellar mobility and different lower body kinematics than patients without PT.

**Background:**

PT is a common overuse condition of the patellar tendon that can cause pain and impair function. Subjects with overuse knee problems display different hip and knee functional mechanics, specifically valgus collapse. Patellar hypermobility has not been specifically studied as a possible risk factor for PT.

**Methods:**

11 patients with PT and 11 controls without PT, age 18 to 40, were studied. Using a patellofemoral arthrometer (PFA), maximal lateral and medial patellar displacement was measured. 3-D motion analysis was performed to determine lower extremity joint motions during single-leg step down and drop vertical jump tests.

**Results:**

Patients with PT had significantly increased lateral patellar mobility compared to controls (12.21 ± 3.33 mm vs. 9.19 ± 1.92 mm, *P* = .017). PT patients showed significantly greater peak hip adduction with both drop vertical jump (2.7° ± 6.3° vs. −5.6° ± 4.2°; *P* = .003) and step down (17.0° ± 3.8° vs. 12.5° ± 4.4°, *P* = .024). PT patients demonstrated increased peak ankle external rotation with drop vertical jump (−21.1° ± 5.9° vs. −14.8° ± 5.5°, *P* = .023) and step down (−15.6° ± 5.5° vs. −9.0° ± 6.0°, *P* = .017).

**Conclusions:**

Patients with PT exhibit increased lateral patellar mobility, hip adduction, and ankle external rotation. The effects of increased patellar mobility deserve further study in the development, management, and prevention of PT.

## 1. Introduction

Patellar tendinopathy (PT), also known as ‘‘jumper’s knee,” describes an overuse condition of the patellar tendon related to inability to the tendon to adapt to loading conditions [1]. PT is commonly seen in clinical practice, with 14% of elite athletes across different sports experiencing the condition. The highest prevalence of PT occurs with athletes in explosive jumping sports such as volleyball (45%) and basketball (32%) [2]. Runners can also get PT with 5–14% of distance runners suffering from PT [2,3]. Symptoms of PT are often prolonged and may lead to reduced training and competition levels and impaired performance levels [2,4]. Development of PT consists of many factors, including intrinsic, anatomical, and biomechanical factors, as well as extrinsic training variables [5].

**Figure 1.**
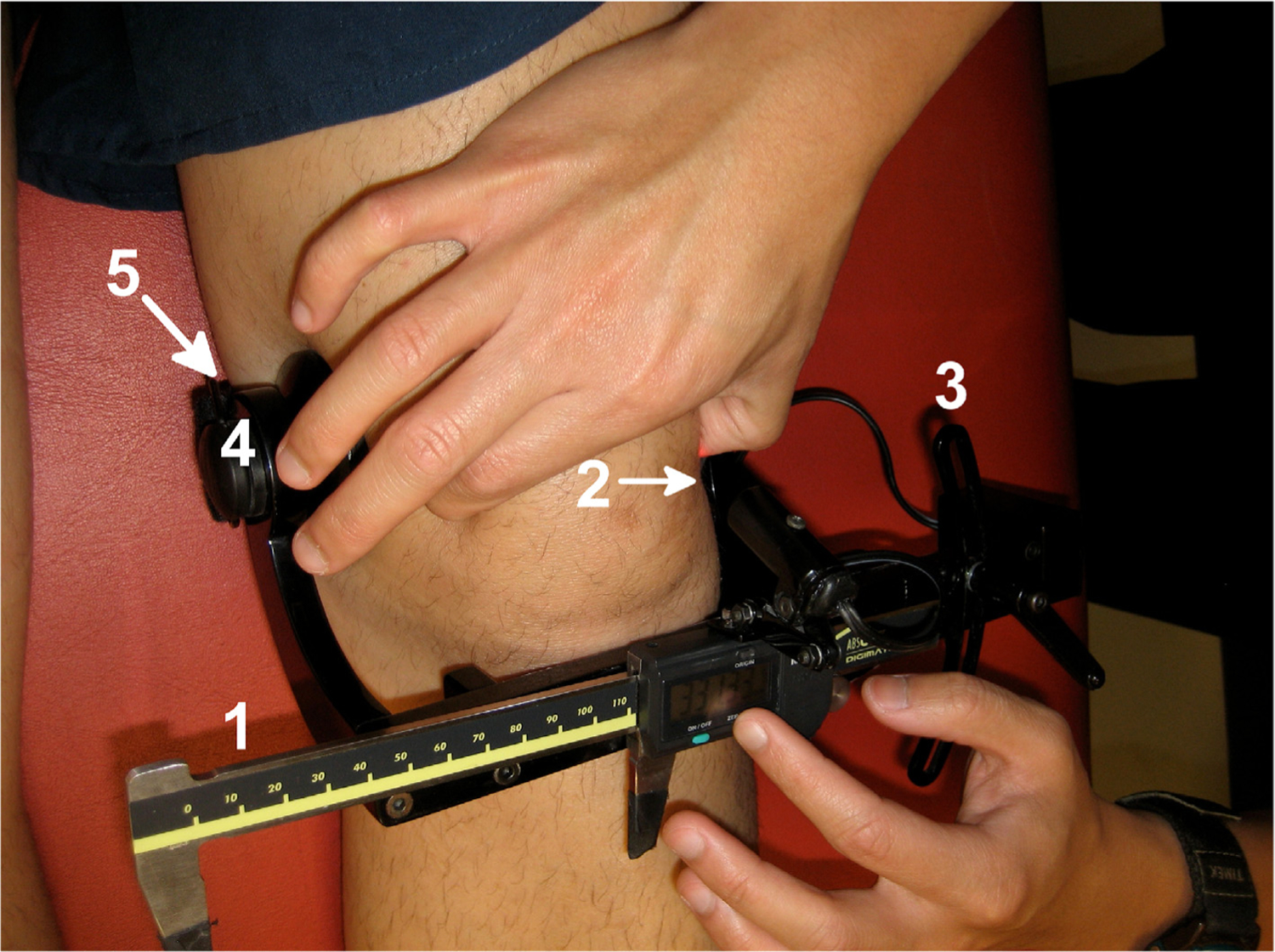
Patellofemoral arthrometer components: [1] digital caliper (ruler) with a precision of 0.01 mm used to obtain quantitative measurements, [2] adjustable laser module arm used to align the medial border of the patella with the ruler, [3] plane adjuster to position the digital caliper, [4] clamping mechanism, and [5] thigh strap used to secure the arthrometer to the patient’s leg.

Patellar mobility has not been measured previously in patients with PT. To measure passive patellar mobility clinically, practitioners often use a manual patellar mobility test [6]. With the lower extremity relaxed and knee flexed to 20 or 30 degrees, the patella is pushed in a medial and lateral direction. The amount of patellar mobility can be expressed in millimeters or typically as a percentage of patellar width, though it is difficult to calculate patellar mobility precisely due to visual assessment and low reliability of these measurement methods. Patellar mobility can be quantified using a device called a patellofemoral arthrometer (Figure 1). The patellofemoral arthrometer has been shown to have good validity comparing arthrometer measurements to those taken from magnetic resonance imaging (MRI) (intraclass correlation coefficient [ICC] 0.86) and excellent intratester (ICC 0.96 and 0.97) and intertester reliability (ICC 0.92) [7,8]. Hypermobility of the joints has been associated with increased knee problems, in particular patellar instability [9]. Abnormal patellar tracking has also been shown to be associated with knee overuse problems, including PT and patellofemoral pain. Allen et al. found patients with proximal PT demonstrated more static lateral subluxation or tilting of the patella using cine-MRI, compared to patients without PT [10]. Souza et al. found that in 15 female patients with patellofemoral pain vs. 15 controls, those with patellofemoral pain demonstrated significantly greater lateral patella displacement at all angles evaluated and significantly greater lateral patella tilt on MRI [11]. Because of the implications of abnormal patellar tracking and mobility on other injuries of the knee, it is important to investigate how patellar mobility may be associated with PT.

There is ample literature that shows that excessive hip adduction is related to lower extremity injuries—for examples, patellofemoral pain [11,12], iliotibial band syndrome [13], and tibial stress fractures [14]. In runners, biomechanical factors that may adversely contribute to PT include earlier knee flexion, earlier internal tibia rotation, and later hip adduction [15]. Biomechanical studies that examined knee and ankle joint dynamics during the volleyball spike jump found that small knee and ankle joint flexion during the first part of landing impact, high knee angular velocities, high range of ankle inversion-eversion moments, high rate of knee moment development, high external tibial rotation and plantarflexion moments, and large vertical ground reaction forces could be linked to the development of PT [16,17].

The aims of this study are to determine: 1) whether patellar mobility measured with a patellofemoral arthrometer is increased in patients with PT, and 2) if there are different lower extremity kinematics, specifically with drop vertical jump and step down task. We hypothesize that patients with PT will display a greater amount of patellar mobility than patients without PT. Also, we hypothesize that patients with PT will have increased hip adduction when compared to controls.

## 2. Methods

### 2.1 Subject recruitment and screening

All subjects were recruited through a university-based sports clinic and underwent a clinical screening exam to determine the presence of PT. The study was approved by the Committee for Human Research at our institution and informed consent was obtained from all participants. Subjects age 18 to 40 were eligible to participate, and PT subjects had to present with maximal symptoms located at the patellar tendon in the affected knee. Control subjects were matched to PT subjects by sex, age, height, mass, and body mass index (BMI) (Table 1). Eleven patients with PT (7 males, 4 females; 8 with unilateral PT, 3 with bilateral PT) and 11 controls (7 males, 4 females) participated. Diagnostic criteria for PT consisted of 1) a history of pain in the proximal patellar tendon in connection with athletic activity and 2) tenderness to palpation of the corresponding area on physical examination [2,18]. Patients with the following conditions were excluded: history of knee surgery including ACL reconstruction, history of knee osteoarthritis, history of lower extremity surgery, or pain primarily caused by a problem other than PT.

### 2.2. Patellofemoral arthrometer measurement

A patellofemoral arthrometer was used to measure lateral and medial patellar mobility for each patient [8]. To measure each patient’s lateral and medial patellar mobility using the arthrometer, the subject was instructed to relax the quadriceps supine on a table with the knee at 0° of flexion, as measured with a standard goniometer. The lower extremity was placed in neutral rotation with a rolled towel placed between the ankles. The ankles were strapped to prevent hip rotation.

The patellofemoral arthrometer was fitted perpendicular in the frontal plane and parallel in the sagittal plane to the long axis of the thigh. The arthrometer was then clamped to the femoral epicondyles and strapped to the thigh. The digital caliper of the arthrometer was adjusted at a 90° angle to a line between the center of the patella and the anterior superior iliac spine (ASIS). To measure the initial position of the patella, the laser of the arthrometer was aligned with the lateral border of the patella. The digital caliper was reset to 0 mm. Lateral patellar mobility was measured by manually pushing the patella laterally with maximum force from the examiner, without causing pain in the subject. The laser was slid along the caliper, and the new position of the lateral patellar border was recorded. Three lateral mobility values were taken for each subject, and the average (mean) value was recorded as the patient’s lateral patellar mobility. Medial patellar mobility was measured in a similar manner, with force on the patella applied in the medial direction. Similarly, three trials were averaged to determine medial patellar mobility. Patellar mobility balance (PMB) was calculated as lateral minus medial patellar mobility [7]. Lateral patellar mobility index and medial patellar mobility index were calculated from the lateral and medial patellar mobility values respectively as a percentage of the subject’s patellar width.

### 2.3. 3-D biomechanical analysis

**Figure 2.**
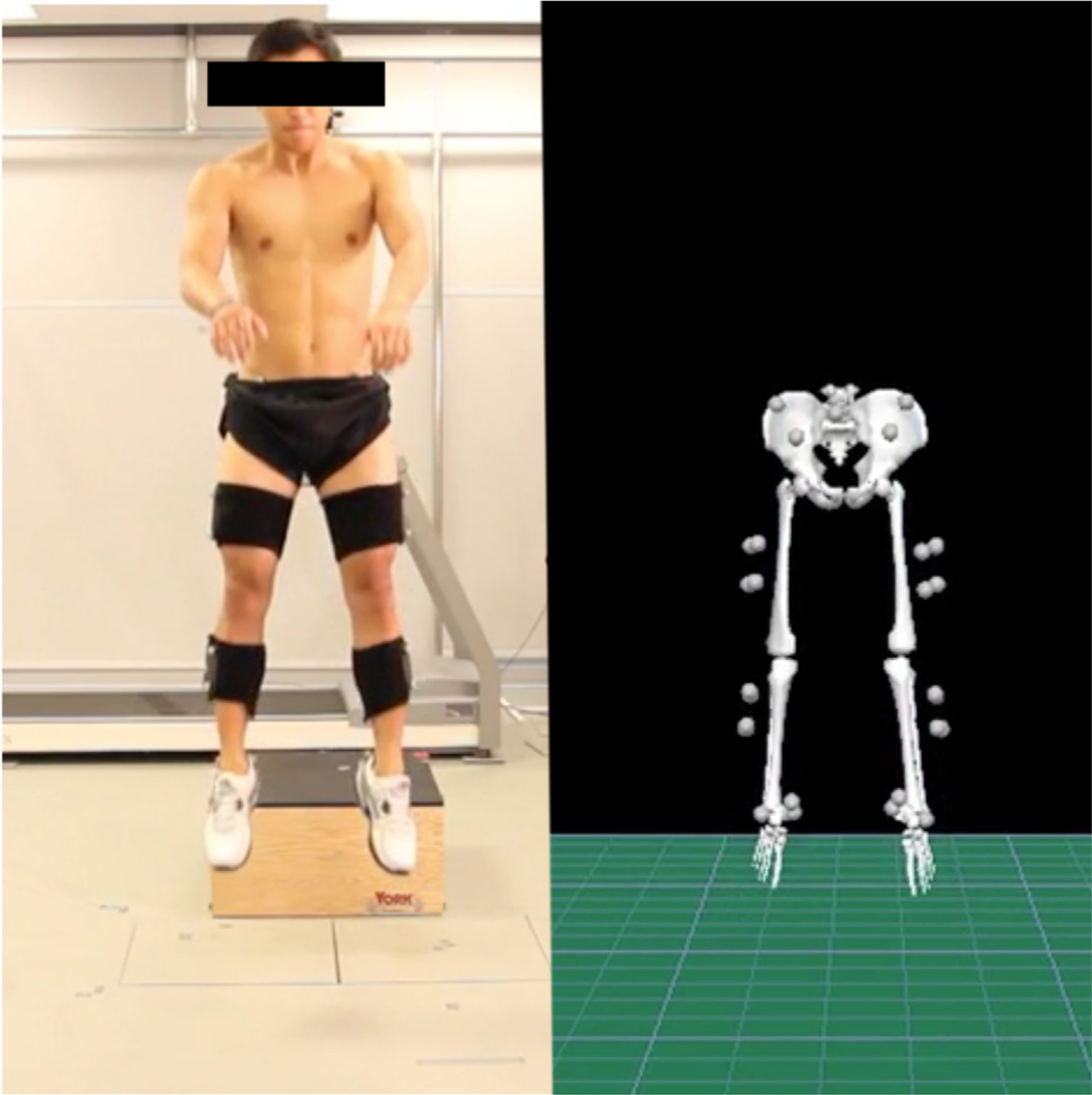
Photo of 3-D motion capture with video image of subject performing a drop jump during the jump phase and corresponding skeletal reconstruction in Visual3D (C-Motion, Inc., Rockville, MD) showing markers.

Three-dimensional motion analysis was performed using a computer-aided video motion analysis system (Vicon, Oxford Metrics LTD. Oxford, UK). Kinematic data was sampled at 100 Hz. Reflective markers (14 mm spheres) were placed over the following bony landmarks: the first and fifth metatarsal heads, medial and lateral malleoli, medial and lateral femoral epicondyles, the joint space between the fifth lumbar and the first sacral spinous processes, and bilaterally over the greater trochanters and iliac crests. Tetrad clusters of rigid reflective markers each were placed on the lateral surfaces of the subject’s thigh and leg, and triad clusters of markers were placed on the lateral surfaces of the heel counter of the shoe [19]. Lower extremity joint motions were measured in the sagittal, frontal, and transverse planes (Figure 2). Kinematics were filtered using a 12 low-pass filter Hz based on results from a residual analysis technique described by David Winter [20]. Joint angles were calculated using a Joint Coordinate System as described by Grood and Suntay [21]. Angles were calculated using a Cartesian Coordinate system as the distal segment moving on the proximal segment. Vicon Nexus software (Vicon Inc,) was used for motion capture, while Visual3D software (C-motion, Georgetown, MD, USA) was used for joint angle calculations. Ground reaction force data was measured using two AMTI force plates (Model #OR6–6-1, Advanced Mechanical Technology, Inc [AMTI], Watertown, Massachusetts) at a rate of 1000 Hz.

Subjects performed two tasks: 1) a single-leg step down test and 2) a drop vertical jump test. A single-leg step down test was performed as has been previously described by Souza and Powers [19]. Subjects stood on top of a 15-cm plyometric step and performed 3 sets of 5 step downs on each leg, for a total of 15 step downs captured per leg. To limit subject fatigue, there was a 1-minute rest period between each of the 3 step down sets and a 5-minute rest period between the step down tests and the drop vertical jump tests. Peak hip, knee, and ankle kinematics in the sagittal, frontal and transverse planes were measured throughout each step down cycle and the drop vertical jump.

For the drop vertical jump test, as described by Hewett et al., subjects stood on top of a 30-cm plyometric step and were instructed to jump off the box, land with each foot on top of its respective force plate, and immediately perform a maximum vertical jump [22]. Three drop vertical jump trials were recorded and analyzed for each subject. Joint angles for the hip, knee, and ankle were measured throughout each drop vertical jump landing and takeoff cycle on the force plate, defined as the point of initial contact with the force plate until takeoff (leaving) the force plate from the vertical jump and includes both the deceleration phase (as the knee flexes) and the acceleration phase (as the knee extends).

### 2.4. Statistical analysis

For subjects with unilateral PT, the affected leg was tested and compared with the same side tested on their matching control subject. For subjects with bilateral PT, the side that was more affected was tested, and the same side on their matching control was tested. All bilateral PT subjects in this study reported that one side was worse than the other. The data were analyzed using SPSS 15.0. Independent two-sample t-tests were used to analyze patellar mobility measurements, as well as joint angles from the biomechanical tests between PT and control patients. Significant difference was set at *P* < .05. Intratester reliability of lateral patellar mobility and medial patellar mobility was assessed by calculating ICCs. The standard error of measurement (SEM) was calculated using the following equation: SEM = SD × √(1 - ICC).

## 3. Results

Subject characteristics are presented in Table 1. No significant differences were observed in age, height, body mass, or BMI. With regard to passive patellar mobility, patients with PT had significantly increased lateral mobility vs. controls (12.2 1 ± 3.33 mm vs. 9.19 ± 1.92 mm); the mean difference between PT subjects and controls was 3.02 mm (95% confidence interval [CI] 0.60 to 5.43 mm, *P* = .017) (Table 2). Lateral patellar mobility index was 21.25 ± 5.74% in PT subjects vs. 15.39 ± 3.02% in controls, with a mean difference of 5.86% (95% CI 1.78 to 9.94%, *P* = .007). There was no difference in average medial patellar mobility or medial patellar mobility index between the groups.

The ICC for intratester reliability of lateral patellar mobility was 0.98 (95% CI 0.95–0.99), with a SEM of 0.48 mm. The ICC for medial patellar mobility was 0.98 (95% CI 0.94–0.99), with a SEM of 0.60 mm.

Peak hip adduction angle was significantly greater in PT patients than controls with both drop vertical jump (2.7° ± 6.3° vs. 5.6° ± 4.2°; *P* = .003) and step down (17.0° ± 3.8° vs. 12.5° ± 4.4°, *P* = .024; Table 3). In the sagittal plane, there were no significant differences between groups, though there was a trend towards increased dorsiflexion of the ankles during both tasks in PT subjects. In the transverse plane, peak ankle angle demonstrated increased external rotation with drop vertical jump (−21.1° ± 5.9° vs. −14.8° ± 5.5°; *P* = .023) and with step down testing (−15.6° ± 5.5° vs. −9.0° ± 6.0°; *P* = .017; Table 3).

## 4. Discussion

We hypothesized that increased mobility of the patella and poor biomechanics are associated with patellar maltracking and instability. Increased patellar mobility was raised as a possible cause for PT as early as 1982 [23] by Roels et al., the same physicians who coined ‘‘jumper’s knee” in 1978 [24]. Our findings showed that patients with PT have more lateral mobility of the patella as well as increased hip adduction and increased ankle external rotation during functional tasks of step down and drop vertical jump. We found significantly increased lateral mobility of the patella in our PT patients, averaging 3.02 mm greater displacement using the arthrometer when compared to controls, which is well outside of our standard error of measurement of 0.48 mm. This translates to a difference of approximately 6% of the patellar width as expressed through the lateral patellar mobility index: 21% of the patellar width laterally in PT subjects compared to 15% in controls. There were no significant differences seen with medial patellar mobility. This is the first report that we are aware of quantifying an increase of lateral patellar mobility using a patellofemoral arthrometer in patients with PT. Our findings are consistent with those of Allen et al. in 1999, who found increased patellar mobility identified by static lateral subluxation or tilting of the patella, using cine-MRI with the knee starting at 25 degrees of flexion moving to full extension [10].

We suggest that under specific biomechanical loads such as jumping sports, the combination of increased lateral patellar mobility with poor proximal hip strength and control during landing may lead to further tensile stresses to the proximal patellar tendon. We found increased hip adduction in PT patients. Our results showed that between PT and control subjects, there was a peak hip adduction difference of 7° for drop vertical jump and 4° for step down. Hip adduction has been implicated in the pathomechanics of other knee conditions, including patellofemoral pain [12] and iliotibial band syndrome [25]. It is likely that the increase in frontal plane hip motion results in altered loads across the knee extensor mechanism, possibly adding strain to particular regions within the patellar tendon. Together, the current results along with these previous studies highlight the clear interdependence of hip and knee mechanics and the likely influence of one joint on proximal and/or distal joints. These findings are important clinically as they represent potential targets for prevention and rehabilitation strategies.

Several papers describe knee flexion to be associated with PT [26,27]. We did not find a significant difference in knee flexion between PT and control subjects; however, we had a small cohort. Future studies should continue to explore both sagittal and frontal/transverse plane kinematics in this population across a range of functional tasks.

PT subjects also demonstrated increased peak external rotation at the ankle compared to controls. The cause of this abnormal motion in the transverse plane remains unclear. There was no significant difference in the landing distance between the knees or the feet between the groups. The PT patients may have an anatomical difference to explain these findings or a compensation effect during step down and drop vertical jump. However, we did not look at segment rotation. We noted non-significant greater sagittal ankle (dorsiflexion) angles in PT subjects compared to controls. Backman et al. found that junior elite basketball players who developed PT had significantly lower mean ankle dorsiflexion range than healthy players. The authors suggested that this lack of dorsiflexion range might lead to compensation in the knee joint and increased load on the patellar tendon [28]. The lack of significant difference which we found might be due to the fact that we measured the angles during dynamic activities, while Backman et al. measured static maximal range of motion. In summary, our findings do show that biomechanical factors at the hip and ankle affect the knee in our PT population differently than in controls. Due to the case-control study design, we cannot demonstrate any cause and effect and recognize that symptomatic patellar tendinopathy could affect an individual’s biomechanics with dynamic testing. Care should be taken in interpreting these findings as the current analysis merely suggests association, and an interventional study would be required to evaluate the causative nature of this relationship.

We propose that patellar mobility may have clinical relevance and should be studied further to identify whether it is a risk factor for patellar tendinopathy. Practitioners can consider examining the knee for increased lateral patellar mobility in the supine position. A single leg flat or preferably an incline squat can assess for hip adduction and abnormal lower limb biomechanics [6,29]. Assessing for increased patellar mobility is also helpful for clinical evaluation of patellar instability which causes other symptomatic issues outside of PT, such as patellofemoral pain and patellar subluxation/dislocation. These are common in pediatric patients who are often more flexible [30].

With hip adduction, valgus collapse of the knee, and increased movement of the patella laterally during flexion, abnormal forces can occur over the proximal tendon, since the patellar tendon is fixed distally. Eccentric exercises, such as decline squats, have played a central role in the treatment of PT [31,32]. These data suggest addressing control of patellar mobility and maltracking as part of managing PT. Specific hip abductor and extension exercises to control hip adduction should be a focus of therapy, in addition to eccentric quadriceps exercises. Hip abduction exercises are successful in assisting in movement patterns for patellofemoral pain patients, which should help in reducing other knee injuries [13]. Investigating whether to use patellar stabilizer braces to reduce the lateral movement of the patella during flexion, as opposed to the traditional patellar strap that is commonly used, is an area for future research.

### 4.1. Limitations and future studies

While static measurement using a patellofemoral arthrometer is one method of evaluating patellar mobility, this method is limited in characterizing the movement of the patella during dynamic tasks. One variable that we did not measure was Q-angle. From our clinical evaluations of the subjects, none of them had any significant static malalignments in the lower extremities. However, since we did not measure Q-angle in these individuals, it is not clear whether this may have contributed to biomechanical outcomes. We did not measure Beighton score for generalized hypermobility, which likely may be related patellar mobility. We also did not measure the Victorian Institute of Sports Assessment (VISA) score which has become a standard for assessing the severity of PT. This limits our ability to compare our study population to other studies. Our small sample size may be underpowered to observe other kinematic variables found positive in other studies. Our selection inclusion criteria are similar to other biomechanical studies, and our patients presented to a sports medicine clinic, so we do not expect our population to have less significant PT problems than other studies. We also did not include any ultrasound or MRI evaluation of the patellar tendon, as the diagnosis is commonly made clinically [18,33]. We did not select subjects based on sex and acknowledge that sex may have an effect on both flexibility and lower extremity biomechanics [19].

Future studies could compare patellar mobility between PT patients and patellofemoral pain patients, since it is not established why certain patients with lower extremity biomechanical problems develop one condition vs. the other. Further studies with PT subjects investigating variables of sex, anatomic lower extremity alignment, and hypermobility are recommended. Animal models or cadavers should be tested to see how the patellar tendon is stressed with lateral patellar shift in addition to tensile load and identify if there is a clinically important amount of lateral patellar mobility that would lead to developing patellar tendinopathy. Assessment of stabilizing the patellar tracking with braces in conjunction with core stability programs for patients with PT can be studied for effects.

## 5. Conclusions

Patients with PT exhibit increased lateral patellar mobility compared to patients without PT. Increased hip adduction and increased ankle external rotation are also associated with patients who have PT. Increased patellar mobility is associated with PT. The effects of patellar mobility deserve further study in the development of PT.

## Figures and Tables

**Table 1:** Subject demographics*.

	PT (n = 11)	Control (n = 11)	Mean difference (95% CI)	*P* Value^†^
Age (in years)	31.7 ± 5.0	30.7 ± 4.5	1.0 (−3.2 to 5.2)	0.627
Height (in cm)	178 ± 6	179 ± 6	0 (−6 to 5)	0.852
Body Mass (in kg)	74 ± 14	78 ± 12	−4 (−16 to 7)	0.453
BMI (in kg/m^2^)	23.0 ± 3.0	24.2 ± 2.6	−1.2 (−3.7 to 1.2)	0.307

Abbreviations: BMI, body mass index; PT, patellar tendinopathy.

* Statistics are listed as mean ± SD.

† *P* values were obtained from independent two-sample t-tests.

**Table 2:** Patellar mobility measurements in PT vs. control subjects*.

	PT (n = 11)	Control (n = 11)	Mean difference (95% CI)	*P* Value^||^
LPM (in mm)	12.21 ± 3.33	9.19 ± 1.92	3.02 (0.60 to 5.43)	0.017
MPM (in mm)	13.70 ± 3.91	13.46 ± 4.12	0.24 (−3.33 to 3.81)	0.891
PMB (in mm)^†^	−1.49 ± 1.93	−4.27 ± 4.47	2.78 (−0.28 to 5.84)	0.073
LPMI (in %)^‡^	21.25 ± 5.74	15.39 ± 3.02	5.86 (1.78 to 9.94)	0.007
MPMI (in %)^§^	23.84 ± 6.78	22.69 ± 7.23	1.16 (−5.08 to 7.39)	0.703

Abbreviations: LPM, lateral patellar mobility; LPMI, lateral patellar mobility index; MPM, medial patellar mobility; MPMI, medial patellar mobility index; PMB, patellar mobility balance; PT, patellar tendinopathy.

* Statistics are listed as mean ± SD.

† PMB = lateral minus medial mobility.

‡ LPMI = (LPM ÷ patellar width) × 100.

§ MPMI = (MPM ÷ patellar width) ×100.

|| *P* values were obtained from independent two-sample t-tests.

**Table 3:** Peak joint angles (in degrees) for step down and drop vertical jump tests in PT vs. control subjects*.

	PT (n = 11)	Control (n = 11)	P Value^†^
Peak sagittal hip angle, drop vertical jump	70.5 ± 21.2	72.5 ± 20.8	0.829
Peak sagittal hip angle, step down	36.3 ± 15.7	34.6 ± 11.4	0.782
Peak frontal hip angle, drop vertical jump	2.7 ± 6.3	−5.6 ± 4.2	0.003
Peak frontal hip angle, step down	17.0 ± 3.8	12.5 ± 4.4	0.024
Peak transverse hip angle, drop vertical jump	2.3 ± 6.4	2.0 ± 7.4	0.941
Peak transverse hip angle, step down	5.0 ± 3.0	6.1 ± 5.6	0.588
Peak sagittal knee angle, drop vertical jump	85.4 ± 8.4	81.3 ± 14.4	0.418
Peak sagittal knee angle, step down	65.5 ± 6.0	64.1 ± 8.2	0.633
Peak frontal knee angle, drop vertical jump	−4.2 ± 5.2	−5.7 ± 8.0	0.621
Peak frontal knee angle, step down	−5.1 ± 3.7	−6.3 ± 5.5	0.570
Peak transverse knee angle, drop vertical jump	0.8 ± 4.7	3.1 ± 6.4	0.362
Peak transverse knee angle, step down	−6.8 ± 4.5	−6.5 ± 6.3	0.928
Peak sagittal ankle angle, drop vertical jump	31.2 ± 5.2	28.0 ± 4.7	0.151
Peak sagittal ankle angle, step down	34.1 ± 3.2	30.9 ± 5.6	0.114
Peak frontal ankle angle, drop vertical jump	−3.2 ± 5.0	−2.2 ± 3.9	0.610
Peak frontal ankle angle, step down	−10.8 ± 2.8	−10.5 ± 2.8	0.812
Peak transverse ankle angle, drop vertical jump	−21.1 ± 5.9	−14.8 ± 5.5	0.023
Peak transverse ankle angle, step down	−15.6 ± 5.5	−9.0 ± 6.0	0.017

Abbreviation: PT, patellar tendinopathy.

* Statistics are listed as mean ± SD. In the sagittal plane, positive angle values denote hip flexion, knee flexion, and ankle dorsiflexion, respectively. In the frontal plane, positive angle values denote hip adduction, knee valgus, and ankle inversion. In the transverse plane, positive angle values denote internal rotation at the hip, knee, and ankle.

† *P* values were obtained from independent two-sample t-tests.
